# Phytogenic compounds do not interfere physiological parameters and growth performances on two Indonesian local breeds of ducks

**DOI:** 10.14202/vetworld.2019.1689-1697

**Published:** 2019-11-04

**Authors:** Ismoyowati Ismoyowati, Diana Indrasanti, Sigit Mugiyono, Mulyoto Pangestu

**Affiliations:** 1 Department of Animal Production, Faculty of Animal Science, Jenderal Soedirman University, Purwokerto, Indonesia; 2 Department of Obstetrics and Gynaecology, Monash University, Clayton, Victoria, Australia

**Keywords:** blood lipid, growth performances, heterophil/lymphocyte ratio, Indonesian ducks, phytogenic compounds

## Abstract

**Aim:**

The present study was to investigate the interaction between duck’s breed and phytogenic compounds as feed additives in the diet on blood lipid and hematological profile, welfare, and growth performance.

**Materials and Methods:**

A total of 200 male day-old local breed ducks (Tegal and Muscovy ducks) were used in this experiment. The first factor was duck breed and the second factor was different phytogenic compounds supplementation in the diet: Garlic, turmeric, ginger, and kencur, at 3% each. The observed variables were the blood lipid profiles comprise high-density lipoprotein (HDL), low-density lipoprotein, cholesterol total, triglyceride, blood parameters, welfare (heterophil/lymphocyte [H/L] ratio), and growth performances (feed consumption, body weight gain, feed conversion ratio, and carcass percentage).

**Results:**

The interaction between breed of ducks and phytogenic compounds had a significant effect on blood triglyceride, but no significant effect on the blood lipid profile, hematological parameters, and growth performances. While, phytogenic compounds in the diet had significant effects on the blood lipid profile, heterophil (H), lymphocyte (L), and H/L ratio of ducks. The breed factors affected HDL and growth performances. Muscovy duck had a higher HDL and growth performance compare to Tegal duck. Among those, garlic most effectively reduced triglyceride level in Tegal duck.

**Conclusion:**

Phytogenic compounds 3% do not have a negative effect on the physiological parameters of ducks increase ducks welfare (H/L ratio), so it does not affect the growth performances of ducks. Muscovy duck had higher growth performances than Tegal ducks.

## Introduction

Duck (*Anas platyrhynchos javanicus*) and Muscovy duck (*Cairina moschata*) are waterfowl, which typically have twice the body fat content of chicken. At present, duck becomes a popular cuisine in Indonesia, its own special class in the society competing with chicken. The population and production of duck meat and egg in Indonesia during 2015 increased by 3.55%, 5.02%, and 3.49%, respectively [1]. Indigenous Indonesian ducks are usually named according to their origin region; they are more adaptable to the tropical environment with optimum productivity. They know as Alabio duck, Magelang duck, Mojosari duck, Tegal duck, Cihateup duck, and Pengging duck. Tegal duck is one of the indigenous Indonesian duck strains, which is maintained around the northern coast of Java. The advantages of Tegal duck are higher egg production, with average duck day production is 70.89% and mature age of 154.5 days [2]. Tegal duck is well adapted in tropical and coastal regions. Adaptable local ducks have strategic position as a source of germplasm as well as a source as a research material [3]. The physical characteristics of Tegal ducks are small head, slim neck, long and round body, wings attached tightly to the body, and the tip of the feathers close above the tail [2]. Muscovy duck (*C. moschata*) is one type of meat-producing domesticated waterfowl for a long time in Indonesia whose wing is shorter than a Pekin duck [4]. It is originated from Central and South America, which was discovered by conquistador from Spain in the early 16^th^ century. Initially, the ducklings were preserved by Colombian, Peruvian, and Brazilian Indians. Some ducks were brought to Europe, Africa, and Asia, including Indonesia. It has a great opportunity to become the source of meat for livestock development [2] because it has high breast meat with unique taste, low fat, savory flavor, and least calorie content than Pekin ducks. Muscovy ducks adapt well to various rearing conditions. It is relatively resistant to the disease and is able to use low-quality feed [5,6]. The purpose of raising Muscovy duck by the rural communities is to make it as an alternative food source and as a parent for incubating duck eggs. Phytogenic compounds are a multifunction feed additive for poultry, including ducks, derived from plant products used in animal feed to improve performance and it enhances immune responses and intestinal quality [7,8]. Phytogenic compounds are primary and secondary plant substances with pharmacological effects and widely used in traditional medicine. Phytogenic compounds are derived from leaves, roots, flowers, and whole plants [9,10]. Most plants contain polyphenol compounds which are classified into flavonoids and non-flavonoids [11] so that feed additives using of phytogenic compounds may improve physiological conditions, immune system, and growth [12]. There are some known phytogenic compounds added in feed such as garlic (*Allium sativum*), turmeric (*Curcuma domestica*), ginger (*Zingiber officinale*), and aromatic ginger (*Kaempferia galanga*, Kencur. Loc.). Garlic is a popular spice for food or medicine against several diseases. It contains an organosulfur thiosulfinates, with allicin as the active component. Allicin (diallyl thiosulfinate) is a key constituent of garlic and has potential health benefit which promotes growth and antioxidant activity including anti-inflammatory, antihypertensive (allicin decreases cholesterol and blood pressure) activities [13,14], and increases production performance [15]. Increasing garlic powder level (5%) in broiler feed lowers total cholesterol and low-density lipoprotein (LDL) and increases high-density lipoprotein (HDL) [16]. Ginger contains several compounds such as gingerol, gingerdiol, and gingerdione that possess strong antioxidant activity [17]. Supplementing 5 g/kg of feed with ginger increases the total activity of superoxide dismutase and glutathione peroxidase but decreases malondialdehyde and cholesterol level in 21- and 42-day-old broilers [18]. Kencur is an important medicinal plant. It has been traditionally used as carminative, cholera, anti-inflammatory, abdominal pain, dyspepsia, and stomach ache as well as in the diseases of coughs, pectoral affections, and stoppage of the nasal blocks [19]. It contains saponin, flavonoid, polyphenol and volatile oil, ethyl-p-methoxycinnamate, and other important compounds of enormous medicinal values and they are very demanding to the traditional health-care practitioner [20].

Bioflavonoid is one of the most varied and vast natural compound groups and tends to be the most significant natural phenol. This compound has a broad spectrum of chemical and biological activity and handles radical [21]. The bioflavonoid modulator effect on cholesterol and lipid has been tested in some animals such as poultry and laboratory animal. Hematological characteristics can be used as an indicator of poultry’s adaptability toward certain environmental conditions. Welfare levels can be measured from the heterophil/lymphocyte (H/L) ratio [22]. Furthermore, H/L ratio has been widely used as a sensitive indicator of long-term stress related to immune function in bird [23]. Active substances in phytogenic compounds have vasorelaxation and antioxidant properties that help animals overcome environmental distress. There are few researches [3,5,6] about Tegal duck and with regard to phytogenic compounds on blood profile and welfare level.

This research was aimed to evaluate the effect of phytogenic compounds on local ducks’ blood lipid profile, welfare level, and growth on two breeds of ducks (Tegal and Muscovy).

## Materials and Methods

### Ethical approval

The experimental protocols were approved by the Animal Ethics Committee, Jenderal Soedirman University No. 1923/UN.23.14/PN.01/2018.

### Preparation of phytogenic compounds powder

Raw material, fresh bulbs of garlic (*A. sativum*) and rhizomes of turmeric (*C. domestica*), ginger (*Z. officinale*), and kencur (*K. galanga*) were obtained from local market in Purwokerto, Indonesia. The raw materials were cleaned, sliced, and washed with sterile distilled water and then oven-dried (55°C) and milled.

### Animals

Hundred day-old male Tegal ducks (*A. platyrhynchos*) weighing 38 g were purchased from duck farmers in the Tegal region and 100 male day-old of Muscovy ducks (*C. moschata*) weighing 40 g were purchased from farmers in the Banyumas area. Each treatment was taken a sample of eight ducks for blood sample collection and slaughter, so the total was 80 ducks. Ducks were kept in brooders from old duck days to 21 days. After the age of 21 days, the ducklings were moved to a colony cage measuring 1×1.5 m, which was filled with five ducklings. Equipment included feeding container, drinking container, digital scale, box, syringe, Vacutainer filled with anticoagulant, and cuvette tube.

### Feeding

Dietary for 1-21 days old was broiler complete feed (Broiler Starter BR I crumble, Japfa Comfeed). Starting from the age of 22 until 70 days, the duck was given experimental feed. The composition of ingredients and nutrient content of the feed is presented in Table-1.

**Table-1:** Ingredient and nutrient composition (g/kg) of diet for ducks (air-dried basis).

Ingredient (kg)	Starter (1-21 days)	Grower/finisher (22-70 days)
Broiler complete feed (BR I)	100	-
Maize	-	40
Rice bran	-	39
Soybean meal	-	12
Fish meal	-	8
Premix (vitamin+mineral)	-	1
Total	100	100
Nutrient content*		
Metabolizable energy (kcal/kg)	3300	2905.45
Crude protein (%)	20.50	16.10
Crude fat (%)	5.00	4.36
Crude fiber (%)	5.70	3.91
Calcium (%)	0.95	1.82
Phosphorus (%)	0.8	1.32

* Proximate analysis

### Phytogenic compounds

Phytogenic compounds were mixed in the basal feed as described in previous studies [13-17]. P0 was basal feed with no phytogenic compounds, P1 (basal feed with 3% garlic), P2 (basal feed with 3% ginger), P3 (basal feed with 3% turmeric) and P4 (basal feed with 3% kencur).

### Husbandry, feeding, and supplementation trial

Up to the age of 21 days, the ducks were kept in a brooder, temperature of 32°C, density of 25-day-old duck (DOD)/m^2^ with 24-h lighting and fed with complete broiler feed. At the age of 22 days, the duck was moved into a colony cage with a density of 5 ducks/1.5 m^2^ with 24-h lighting. Phytogenic compounds were given to the ducks from day 22 to 70. The feed and drinking water was given *ad libitum*.

### Experimental design

The completely randomized factorial design with two factors. The first factor was Breed (B)B1 Tegal ducks (*A. platyrhynchos*) and B2 was Muscovy ducks (*C. moschata*). The second factor is Phytogenic compounds (P).P0 = control feed (basal feed only), P1 = basal feed+3% garlic powder, P2 = basal feed+3% curcuma powder, P3 = basal feed+ginger powder 3%, and P4 = basal feed+kencur powder 3%. Observation were made on blood lipid profiles comprises cholesterol level, HDL, LDL, blood triglycerides and H/L ratios, growth, body weight, and carcass percentage.

### Blood sample collection, analysis, measurement of growth, and carcass percentage

Blood sample was collected at day 70 from brachial vein of duck using a sterilized syringe and needles then transferred into vacutainer tubes with anticoagulant ethylenediaminetetraacetic acid. Blood samples were centrifuged at 2000× *g* for 30 min. Blood plasma was collected and stored at −20°C for further assay of total cholesterol, LDL, HDL, and triglyceride using a spectrophotometer at a wavelength of 500 nm and 340 nm using Hitachi 704 analyzer [24].

Hematological profiles were measure according to Hrabčáková *et al.*, 2014 [25]. The number of red blood cells (RBCs; million/ml) was measured using a hemocytometer with the Hayem dilution method. Hematocrit or packed cell volume was determined by the microcapillary reader by a microhematocrit centrifuge with a maximum relative centrifugal force of 10,000× *g*, which should be reached within 30 s; then, the hematocrit value was indicated on the microcapillary reader.

The number of white blood cells (WBCs) and differential leukocytes performed using blood smears stained with Wright method, using a Wright Stain Hema-Tek Stain (WSHT, Sigma Aldrich, St. Louis, MO). One hundred WBCs per blood sample were examined using a microscope (Eclipse 100, Nikon, Japan) with 400×, as well as the identification heterophils, lymphocytes, monocytes, eosinophils, and basophils. The total heterophil and eosinophil count were calculated using a Neubauer hemacytometer, with phloxine B cell staining using water propylene glycol. The total number of lymphocytes, monocytes, and basophils was determined indirectly by calculation of the percentage and total heterophils cells and eosinophils.

The concentration of hemoglobin (g/100 ml), total plasma protein (TPP), and blood albumin were measured by spectrophotometry [26]. The total cholesterol, HDL cholesterol, triglycerides, and LDL cholesterol were measured using Hitachi 704.

### Body weight and food consumption

The measurements of body weight and feed consumption were carried out every week for 10 weeks, starting from DODs. Feed conversion is calculated based on the amount of feed consumed for 10 weeks divided by body weight gain during 10 weeks of maintenance of ducks. At the age of 10 weeks, each sample unit was taken from two ducks from 10 treatments, to be slaughtered. Each treatment was four replications so that a total of 80 ducks were slaughtered for the measurement of carcass production. The carcass weight is the weight without blood, feather, head, neck, shank, and internal organs (liver, gizzard, intestine, and heart). The carcass percentage is the carcass weight compared to gross body weight after slaughter.

### Statistical analysis

Data were analyzed using Systat 13 (Systat Software, Inc., San Jose, CA). Data were subject to analysis of variance (ANOVA) according to factorial randomized complete design. Significant differences between treatment means were determined using a Duncan multiple range test. Statements of significance were based on p<0.05.

## Results

### Blood profiles

The result of blood lipid profiles consists of total cholesterol, LDL, HDL, and triglyceride is presented in Table-2. Results of ANOVA show interaction between breed and phytogenic compounds. Triglyceride level in the blood was significantly affected (Table-2). Group of Tegal duck with garlic powder supplement had the lowest blood triglyceride compared to groups of Muscovy ducks. Turmeric powder supplement had a relatively similar effect on both Tegal and Muscovy ducks. Garlic was the most effective phytogenic to lower blood cholesterol levels and LDL while increasing HDL (Table-3).

**Table-2:** Effect of interaction between breeds and phytogenics supplement on blood lipid profile.

Treatments	Cholesterol (mg/dl)	HDL-C (mg/dl)	LDL-C (mg/dl)	Triglyceride (mg/dl)
B1P0	250.00±21.28	52.80±3.59	197.20±23.04	187.50±15.96^a^
BIP1	175.00±13.98	93.50±7.51	81.50±17.58	108.33±21.52^d^
B1P2	186.11±10.64	85.25±5.50	100.86±9.07	129.17±34.36^c^
B1P3	222.22±18.14	64.35±4.88	157.87±22.72	187.50±15.96^a^
B1P4	238.89±37.95	61.60±9.51	177.29±43.09	237.50±55.07^a^
Average B1	214.44±36.07	71.50±16.17^b^	142.94±51.02	170.00±55.30
B2P0	247.22±13.98	74.25±6.81	172.97±18.32	216.67±23.57^a^
B2P1	177.78±18.14	100.10±8.33	77.68±19.96	154.17±25.00^b^
B2P2	177.78±18.14	92.40±9.51	85.38±26.08	145.83±36.96^c^
B2P3	225.00±18.98	67.65±7.49	157.35±23.64	183.33±13.61^a^
B2P4	244.44±18.14	62.70±5.54	181.74±22.14	170.83±28.46^b^
Average B2	214.44±35.34	79.42±16.29^a^	135.02±49.67	174.00±34.82

B1P0=Tegal ducks fed with basal feed, B1P1=Tegal ducks fed with basal feed+3% garlic powder, B1P2=Tegal ducks fed with basal feed+3% turmeric powder, B1P3=Tegal ducks fed with basal feed+3% ginger powder, and B1P4=Tegal ducks fed with basal feed+3% kencur powder, B2P0=Muscovy ducks fed with basal feed, B2P1=Muscovy ducks fed with basal feed+3% garlic powder, B2P2=Muscovy ducks fed with basal feed+3% turmeric powder, and B2P4=Muscovy ducks fed with basal feed+3% kencur powder, B1=Tegal duck, B2=Muscovy duck.

a,b,c,d Means within a column with uncommon letters differ at p<0.05. HDL-C=High-density lipoprotein cholesterol, LDL-C=Low-density lipoprotein cholesterol

**Table-3:** Effect of phytogenics supplement on cholesterol level.

Phytogenics	Cholesterol (mg/dl)	HDL-C (mg/dl)	LDL-C (mg/dl)
Control	248.61±16.73^a^	63.53±12.52^c^	185.09±23.22^a^
3% Garlic	176.39±15.07^d^	96.80±8.15^a^	79.59±17.53^d^
3% Turmeric	181.94±14.47^c^	88.83±8.14^b^	93.12±19.88^c^
3% Ginger	223.61±17.25^b^	66.00±6.11^c^	157.61±21.46^b^
3% Kencur	241.67±27.70^b^	62.15±7.23^c^	179.52±31.80^b^

a,b,c,d Means within the same column with uncommon superscript differ significantly (p<0.05). HDL-C=High-density lipoprotein cholesterol, LDL-C=Low-density lipoprotein cholesterol

Variety of phytogenic compounds has no effect on RBC, hematocrit, hemoglobin, albumin levels, and fibrinogen (Table-4) on both breeds. These results indicate that phytogenic compounds do not interfere with the physiological condition. However, ANOVA showed a significant interaction between breeds and various phytogenic compounds on TPP. Animal with ginger supplementation showed higher TPP. Muscovy ducks that were fed supplemented with ginger powder had lower TPP than the control and turmeric supplementation. ANOVA results show that supplementing phytogenic compounds significantly affected the H/L ratio as a stress indicator and body immunity of fowl (Table-5). All phytogenic compounds lowered H/L ratios compared to the control group.

**Table-4:** Effect of interaction between breeds and phytogenics supplement on hematological parameters.

Treatments	RBC (mill/ul)	WBC (cells/ul)	Hb (mg/dl)	PCV (%)	TPP (g/dl)	Albumin (g/dl)	Fibrinogen (g/dl)	Heterophil (%)	Lymphocyte (%)	Monocyte (%)	Eosinophil (%)	H/L
B1P0	3.27±0.31	11,212.50±2259.93	12.99±1.10	36.00±5.89	4.49±0.51	6.34±0.93	1.93±0.70	51.25±6.85	31.75±9.22	4.50±1.73	12.50±3.42	1.93±0.70
BIP1	3.31±0.24	7887.5±2930.41	13.5±1.05	42.00±4.08	2.75±0.19	6.98±1.67	0.57±0.11	29.25±2.99	55.00±2.83	4.75±1.74	11.00±2.45	0.57±0.11
B1P2	2.83±0.81	10,687.50±3306.15	12.18±1.65	37.50±7.55	4.05±1.15	6.59±1.15	1.00±0.30	39.00±7.53	40.00±5.42	4.00±1.41	17.00±6.22	1.00±0.30
B1P3	3.21±0.40	10,787.50±2342.50	12.68±1.59	43.75±4.79	4.85±0.77	8.36±1.50	0.81±0.18	37.00±5.35	46.25±4.19	5.00±0.82	11.75±3.30	0.81±0.18
B1P4	3.04±0.36	9509.25±2383.17	12.19±0.86	39.94±5.60	4.41±0.74	7.80±0.91	1.31±0.81	43.50±11.09	38.00±10.68	5.75±3.59	12.75±2.99	1.31±0.81
B2P0	3.10±0.44	9409.38±1768.34	12.52±1.35	42.44±9.19	4.20±0.37	8.58±1.87	1.67±0.59	51.50±8.54	33.00±6.58	4.75±1.50	10.75±2.63	1.67±0.59
B2P1	3.11±0.57	7600.00±1918.33	12.28±1.24	42.50±7.72	2.90±0.12	6.25±0.87	0.57±0.27	31.75±10.34	58.25±6.99	4.50±1.29	8.00±2.83	0.57±0.27
B2P2	2.88±0.70	10,500.00±2920.05	12.98±1.58	41.75±9.18	4.85±0.62	8.66±1.28	1.12±0.62	39.50±11.50	40.50±12.77	4.75±1.50	15.25±3.77	1.12±0.62
B2P3	3.14±0.57	8500.00±2171.02	12.75±0.85	42.50±7.72	3.50±0.81	7.93±1.67	0.69±0.18	34.75±4.99	52.00±7.70	5.00±1.41	8.25±2.63	0.69±0.18
B2P4	3.02±0.43	8825.00±2915.90	11.83±1.00	41.00±7.39	3.00±0.91	6.98±1.14	1.12±0.64	41.00±10.55	42.75±14.10	4.00±1.41	12.25±3.86	1.12±0.64

B1P0=Tegal ducks fed with basal feed, B1P1=Tegal ducks fed with basal feed+3% garlic powder, B1P2=Tegal ducks fed with basal feed+3% turmeric powder, B1P3=Tegal ducks fed with basal feed+3% ginger powder, and B1P4=Tegal ducks fed with basal feed+3% kencur powder, B2P0=Muscovy ducks fed with basal feed, B2P1=Muscovy ducks fed with basal feed+3% garlic powder, B2P2=Muscovy ducks fed with basal feed+3% turmeric powder, and B2P4=Muscovy ducks fed with basal feed+3% kencur powder. ^a,b,c,d^Means within a column with uncommon letters differ at p<0.05. RBC=Red blood cell, WBC=White blood cell, PCV=Packed cell volume, TPP=Total plasma protein, H/L=Heterophil/lymphocyte

**Table-5:** Effect of phytogenics supplement on heterophils, lymphocytes, and H/L.

Phytogenics	Heterophils (%)	Lymphocytes (%)	H/L
Control	51.38±7.17^a^	32.38±7.44^c^	1.80±0.62^a^
3% Garlic	30.50±7.17^c^	56.63±5.24^a^	0.57±0.19^b^
3% Turmeric	39.25±9.00^b^	40.25±9.08^b^	1.06±0.46^b^
3% Ginger	35.88±4.94^b^	49.13±6.51^a^	0.75±0.18^b^
3% Kaempferia	42.25±10.11^b^	40.38±11.86^b^	1.21±0.68^b^

a,b,c,d Means within the same column with uncommon superscript differ significantly (p<0.05)

### Body weight gain, feed conversion ratio (FCR), and carcass percentage

The results showed the interaction between ducks breed and phytogenic compounds, as well as phytogenic compounds had no significant effect (p>0.05) on feed consumption, body weight gain, FCR, and carcass percentage of duck for 10 weeks of maintenance. On the other hand, ducks breed alone has a significant effect (p<0.05) on feed consumption, body weight gain, FCR, and the carcass percentage of duck. The average body weight gain, FCR, and carcass percentage of duck due to phytogenic compounds are presented in Table-6. Feed consumption, body weight gain, and carcass percentage for Tegal duck were lower than Muscovy ducks. This indicates that Muscovy duck was more efficient in feed, due to its higher FCR than Tegal duck.

**Table-6:** Effect of interaction between breeds and phytogenics supplement on feed consumption, body weight, feed conversion ratio, and carcass percentage.

Treatments	Feed consumption (g/10 weeks)	Body weight gain (g)	Feed conversion ratio	Carcass percentage (%)
B1P0	4758.78±25.80	1489±14.47	3.20±0.04	56.50±1.42
B1P1	4731.72±48.14	1481.75±75.71	3.22±0.33	56.63±1.45
B1P2	4762.86±27.67	1409.75±59.50	3.38±0.02	55.13±3.41
B1P3	4752.36±21.78	1484.10±61.79	3.21±0.13	56.62±1.56
B1P4	4697.68±75.22	1499.15±68.51	3.14±0.13	55.05±1.42
Average B1	4740.68±45.78^b^	1472.75±88.84^b^	3.23±0.18^a^	55.99±1.94^b^
B2P0	5312.09±155.86	1872.50±78.44	2.84±0.12	59.27±1.36
B2P1	5173.94±112.13	1998.00±59.27	2.59±0.07	58.69±1.35
B2P2	5240.63±147.31	1974.11±96.10	2.66±0.11	56.18±0.82
B2P3	5178.69±82.63	2029.96±171.52	2.54±0.26	57.87±1.73
B2P4	5190.56±161.23	2017.85±149.51	2.58±0.24	57.76±1.44
Average B2	5219.18±123.83^a^	1978.48±120.38^a^	2.64±0.19^b^	57.95±1.62^a^

B1P0=Tegal ducks fed with basal feed, B1P1=Tegal ducks fed with basal feed+3% garlic powder, B1P2=Tegal ducks fed with basal feed+3% turmeric powder, B1P3=Tegal ducks fed with basal feed+3% ginger powder, and B1P4=Tegal ducks fed with basal feed+3% kencur powder, B2P0=Muscovy ducks fed with basal feed, B2P1=Muscovy ducks fed with basal feed+3% garlic powder, B2P2=Muscovy ducks fed with basal feed+3% turmeric powder, and B2P4=Muscovy ducks fed with basal feed+3% kencur powder, B1=Tegal duck. B2=Muscovy duck.

a,b Means within the same column with uncommon superscript differ significantly (p<0.05)

## Discussion

### Blood profiles

Tegal duck is an Indonesian indigenous duck that possesses unique adaptive traits that permit them to survive and reproduce under harsh climatic, nutritional, and management conditions. Muscovy duck is a waterfowl that originated from Latin America, is now well adapted to the Indonesian climate that adapted for survival under scavenging free-range conditions due to their involvement from the same conditions and beneficial to rural people because they are available, adaptable [4].

Research about herbal plant has been done [13,27], but the research of phytogenic supplementation on waterfowl in Indonesia is still limited. The previous research indicated that phytobiotic properties of garlic (*A. sativum*), turmeric (*C. domestica*), red ginger (*Z. officinale*), and kencur (*K. galanga*) were studied using standard *in vitro* antibacterial test and *in vivo* feeding trial with ducklings. Weight gain, feed intake, and FCR of ducklings were affected by inclusion of garlic, red ginger, and kencur [28] and substituted with antibiotics in the feed of broiler [29]. The highest antibacterial activity against Salmonella pullorum and *Escherichia coli* was observed with garlic; it was also significantly effective against *Clostridium perfringens* [30].

Genetic factors influence blood lipid profile on ducks. Both ducks are waterfowl with high subcutaneous fat. Tegal ducks are laying type ducks, with lower body weight compared to Muscovy ducks. Genetically, both total cholesterol and HDL in the blood are determined by endogenous factors and the feed consumed by ducks (Table-2).

There is a different breed response on phytogenic supplementation. Garlic reduced blood triglycerides in Tegal duck, while turmeric is more effective in lowering blood triglycerides levels in Muscovy (Table-2). Result in Tables-2 and 3 agreed with the previous research that supplementing 5% garlic powder or 3% garlic powder plus α-tocopherol resulted in significantly lower total and LDL cholesterol levels and greater HDL cholesterol levels compared with the control [16]. Triglyceride, the precursor for energy is the most abundant energy forms stored in the body. When the body needs energy, the lipase enzyme in lipid cells will break the triglycerides into glycerol and fatty acid then release them into the arteries. Fatty acid then undergoes hydrolysis in the liver, yielding by-products such as cholesterol.

Garlic was the most effective agent to reduce blood lipid. About 3% garlic powder in diet have a much higher antioxidant capacity or biological effects in suppressing the formation of free radicals [31]. Administrating garlic powder decreased the blood LDL and non-esterified fatty acids without affecting the blood triglyceride, HDL, and beta-hydroxybutyric acid concentrations [32]. Turmeric powder (*C. domestica* Val.) up to 0.6% increased the number of erythrocytes, hemoglobin concentration, hematocrit, and total lymphocyte percentage, while the number of heterophils, monocytes, eosinophils, and basophils decreased in duck [33].

All types of phytogenics showed a relatively similar effect as hematologic profile, stress, and welfare level modulators in two Indonesian local breeds of ducks (Table-4). Harper *et al*. [34] indicated that bioactive substances; allin, allicin, curcumin, bioflavonoid in ginger, and kencur, serve as antioxidants in the immunomodulatory system. Differences in the responses to ginger on Tegal ducks and Muscovy ducks show that physiological levels of TPP will adjust the protein requirements in the tissues. The plasma levels of total protein are 3.2-5.6 g/dL, albumin ranges from 52% to 65%, and globulin from 29.5% to 54%. In normal conditions, the albumin and globulin ratio is 1.2:1. TPPs can function as the body’s protein reserves. Circulating plasma proteins are not static, they are constantly held in exchanges with tissue backup labile, where the number is proportional to the circulating protein resulting in a dynamic balance. At a time of protein deficiency, the body takes up a network of proteins and plasma proteins for metabolic needs [34].

Welfare levels can be measured from the H/L ratio [22]. Bioactive substances in garlic, ginger, and turmeric have similar effectiveness to promote the immune system (Table-5). The lymphocyte is one type of agranulocyte leukocyte with predominant percentage in fowl blood. The lymphocyte amount reflects body immunity in that the less lymphocyte the weaker the body immunity. Active substances in phytogenics have vasorelaxation and antioxidant properties that help animals overcome environmental distress. Distressed fowl will undergo lymphocyte decrease and heterophil increase, thereby increasing the H/L ratio [35]. The lymphocyte proliferative response is commonly used to assess the immunomodulatory effects of a potential therapeutic agent. The medical effects of garlic and its content have been widely investigated. Biological responses to garlic are shown to lower cancer and antitumor risks and stimulate immune function [36]. Some research reported that curcumin has been investigated for its effects on the mitogen-induced proliferation of T-cells *in vitro* and *in vivo*; curcumin reasonably boosts the number of T- and B-cells without changing the number of phagocytes and macrophages [37]. Curcumin is an effective immunomodulatory agent that can alter the activation of T- and B-cells [38].

Kencur shows antioxidant activity that can decrease free radical activity and oxidation of various molecules and may possibly have health-promoting special effects in the prevention of degenerative diseases [39]. This antioxidant activity is mainly due to the total phenolic content and flavonoids, including luteolin and epidenin [40]. Kaempferia supplementation can lower stress in ducks because the ethyl cinnamate contained in it has vasorelaxant activity [41]. Ethyl cinnamate leads to the inhibition of calcium influx into vascular cells and releases nitric oxide and prostaglandins from endothelial cells [42]. Ginger suppressed lymphocyte proliferation; this was mediated by decreases in interleukin (IL)-2 and IL-10 production [43]. Another study found an aqueous ginger extract significantly increased the production of IL-1β, IL-6, and tumor necrosis factor-alpha in activated peritoneal mouse macrophages [44].

### Body weight gain, FCR, and carcass percentage

Phytogenic supplementation had no significant effect (p>0.05) on feed consumption, body weight gain, feed conversion, and carcass percentage of duck. Garlic, turmeric, ginger, and kaempferia powder provide relatively the same effect as the control. Breed of duck had a significant effect (p<0.05) on feed consumption, body weight gain, feed conversion, and carcass percentage of duck.

The results showed that Muscovy produced a higher body weight gain and carcass percentage and lower feed conversion (p<0.05). Tegal and Muscovy ducks are waterfowl which have different growth characteristics (Table-6). The Tegal duck (*A. platyrhynchos*) is commonly bred for egg production in Indonesia. On the other hand, Muscovy duck (*C. moschata*) was meat type and has high breast meat production. Tegal duck had economic traits such as carcass traits and growth performance was lower than Muscovy duck, which is consistent with the previous studies [6]. The carcass traits and growth performance are very significant in duck production. These traits are controlled by sets of candidate genes which play an important role in ducks growth and development as the insulin-like growth factor genes (IGF-1). The IGF-1 has the potential to be a key regulator of growth, body composition, and skeletal traits during postnatal development. The Muscovy ducks showed higher significant IGF-1 gene expression followed by Mulard and Pekin ducks in that order [6]. Muscovy duck is a waterfowl with specific characteristics of sex dimorphism. The male Muscovy has a higher growth rate and carcass production, at the same age, male Muscovy ducks produce body weight gain and relative growth is higher than female ducks. The highest body weight increases in Muscovy ducks at 3 weeks of age, the growth rate begins to decline at the age of 4 weeks [45].

The carcass percentage of Muscovy duck was higher because body weight and breast and thigh meat gain are higher than Tegal ducks. High growth in Muscovy ducks is partly due to the regulation of growth of the hormone IGF. The IGF is an important regulatory system for controlling the development and growth of mammals and poultry. IGF-1, as a member of the IGF family, is a hormone that regulates growth, metabolism, body composition, skeletal characteristics, fat deposition, and growth [46]. Meanwhile, IGF receptor (IGF1R) is a membrane glycoprotein that mediates the biological action of IGF-1 and IGF-2, which has a large effect on chicken growth and the quality of meat and carcass. The IGF1R plays an important role at the developmental and adult stages such as cell cycles, transplants, subsistence, metabolism, multiplication, and cell differentiation [47]. In the previous studies, higher hepatic IGF-1 expression showed breed specificity in ducks [46] and chickens [48]. Significant differences in IGF-1 expression among various ducks (Muscovy, Pekin, and Mule) showed the highest expression observed in Muscovy, this indicates that IGF-1 is responsible for higher muscle growth and carcass in Muscovy ducks [49]. The Muscovy ducks produced body weight gain and carcass production, higher than Tegal ducks, also due to higher feed consumption, and were more efficient with low FCR.

## Conclusion

Phytogenic compounds as feed additives as much as 3% do not have a negative influence on growth performance, carcass production, and physiological conditions of ducks, it can even reduce blood lipid profiles and improve duck welfare. Muscovy duck had a higher growth rate and carcass percentage than Tegal ducks. It is, therefore, recommended for further research on phytogenic compounds on the quality of meat, especially the meat lipid profile.

## Authors’ Contributions

II conceived and designed the study and performed the statistical analyses. DI and SM collected samples and performed the experiments and the laboratory analyses. II, MP, and DI drafted the manuscript and revised the manuscript critically. All authors read and approved the final manuscript.
